# Psychometric properties of the Spanish version of the Jefferson Scale of Empathy: making sense of the total score through a second order confirmatory factor analysis

**DOI:** 10.1186/s12909-016-0763-5

**Published:** 2016-09-19

**Authors:** Alexandra Ferreira-Valente, Patrício Costa, Marta Elorduy, Montserrat Virumbrales, Manuel J. Costa, Jorge Palés

**Affiliations:** 1 University of Minho, School of Health Sciences, Life and Health Sciences Research Institute, Campus de Gualtar, 4710-057 Braga, Portugal; 2 Universitat Internacional de Catalunya, School of Medicine and Health Sciences, Josep Trueta s/n 08165 Sant Cugat del Vallès, Barcelona, Spain; 3 University of Barcelona, School of Medicine, Casanova 143, 08036 Barcelona, Spain

**Keywords:** Empathy, Medical students, Validity and reliability, Second order confirmatory factor analysis, Gender

## Abstract

**Background:**

Empathy is a key aspect of the physician-patient interactions. The Jefferson Scale of Empathy (JSE) is one of the most used empathy measures of medical students. The development of cross-cultural empathy studies depends on valid and reliable translations of the JSE. This study sought to: (1) adapt and assess the psychometric properties in Spanish students of the Spanish JSE validated in Mexican students; (2) test a second order latent factor model.

**Methods:**

The Spanish JSE was adapted from the Spanish JSE-S, resulting in a final version of the measure. A non-probabilistic sample of 1104 medical students of two Spanish medical schools completed a socio-demographic and the Spanish JSE-S. Descriptive statistics, along with a confirmatory factor analysis, the average variance extracted (AVE), Cronbach’s alphas and composite reliability (CR) coefficients were computed. An independent samples *t*-test was performed to access sex differences.

**Results:**

The Spanish JSE-S demonstrated acceptable to good sensitivity (individual items – except for item 2 – and JSE-S total score: −2.72 < *Sk* < 0.35 and −0.77 < *Ku* < 7.85), convergent validity (AVE: between 0.28 and 0.45) and reliability (Cronbach’s alphas: between 0.62 and 0.78; CR: between 0.62 and 0.87). The confirmatory factor analysis supported the three-factor solution and the second order latent factor model.

**Conclusions:**

The findings provide support for the sensitivity, construct validity and reliability of the adapted Spanish JSE-S with Spanish medical students. Data confirm the hypothesized second order latent factor model. This version may be useful in future research examining empathy in Spanish medical students, as well as in cross-cultural studies.

**Electronic supplementary material:**

The online version of this article (doi:10.1186/s12909-016-0763-5) contains supplementary material, which is available to authorized users.

## Background

Empathy has long been considered a key aspect of the therapeutic alliance, and of optimal care [1]. The concept was first introduced by Robert Vicher in 1872 [2], and has merited the attention of clinicians and researchers. The crucial role of empathy in the patient-therapist relationship was pointed by Carl Rogers, who considered empathy as the ability to “perceive the internal frame of reference of another with accuracy *as if one were* the other person but without ever losing the ‘as if’ condition” (p. 210) [3]. Rogers underlined the cognitive dimension of empathy, and stressed that empathy is an indispensable condition to the self-actualization and personal growth of the patient.

Ever since Fine and Therrien [4] studied empathy in the context of physician-patient interactions, clinicians and researchers became increasingly interested in empathy in the context of patient care. (see Hojat, 2007 for a comprehensive review [5]). Empirical findings consistently associate empathy with improved accurateness and celerity of diagnosis, patient’s adherence to treatment, better quality of life and well-being [5–8]. The importance of empathy is thus, generally recognized [9] and international recommendations for medical education highlight the need for understanding and developing it in physicians and in medical students [10–12].

There are multiple and often contradictory definitions for the construct of empathy [9]. The inexistence of a consensual definition translates into the co-existence of more than 40 empathy measures [13], that reflect conceptions of the construct as predominantly cognitive [2, 14–16], affective [17, 18] or both [19, 20]. Culture also influences the meanings that people impart to empathy [5, 21, 22]. The elucidation of cross-cultural differences and similarities related with empathy development during medical training and with the way students and clinicians conceive and manifest empathy would benefit from conceptual clarification of the construct and the application of valid and reliable empathy measures across countries.

One important contribution to the establishment of a widely accepted empathy definition and measure was given by Hojat and colleagues [2]. The authors developed an empathy self-report measure specifically designed to assess physicians’ and medical students’ attitude towards empathy in patient care [23]. The Jefferson Scale of Physician Empathy – Students version (JSE-S) reflects Hojat’s and colleagues [2, 5, 23] definition of empathy as a predominantly cognitive attribute (as opposed to sympathy) involving the ability to understand the patient’s perspective and inner experiences, and the capacity to communicate it. This definition settles in a tripartite view of the construct (attested by the factorial analysis of the JSE-S) comprising the ability to take the patient’s perspective (perspective taking), to stand in the patient’s shoes (standing in the patient’s shoes), and of combining empathy with a sufficient degree of sympathy (compassionate care) [2, 23, 24]. The JSE-S is currently one of the most commonly used measures in research in medical education worldwide. The measure has proved to have adequate validity and reliability across multiple countries and languages [2, 22, 23, 25–33]. The three factor solution found in the original version [2, 23] has been supported in subsequent studies with the original and the translated versions (cf. Table 1). Yet, different factor structures have also emerged. For example, the exploratory factor analysis yielded a five and four factors solution in the Japanese’s and German’s versions [22, 30], respectively.

**Table 1:** Summary of previous research on JPSE-S psychometric properties

Reference	Country	N	PCA (Varimax rotation)	CFA (Maximum likelihood estimation)	Reliability and Convergent Validity	M vs F
Alcorta-Garza et al. (2005) [25]	Mexico	1022	3 factors: PT (10items), CC (7 items), SPS (3 items) Variance explained: not reported Factor loadings > 0.30 (except for Item 18)	-	α = 0.74	M < F
Costa et al. (in press)	Portugal	979	-	Modified model: *χ* ^2^/df = 3.36; CFI = 0.89; PCFI = 0.78; GFI = 0.94; PGFI = 0.75; RMSEA = 0.05 (n.s.); ECVI = 0.66 Saturation levels > 0.30 (except for item 18 and 19) *r* between factors: 0.07 ≤ *r* ≤ 0.72	α JSE-S:0.78 PT:0.76 CC:0.62 SPS:0.62 CR JSE:0.87 PT:0.79 CC:0.67 SPS:0.62 AVE PT: 0.36 CC: 0.29 SPS: 0.59	-
Kataoka et al. (2009) [22]	Japan	400	5 factors Variance explained: 53 % Factor loadings > 0.30 Items load in different factors comparing to the original JSE-S	-	α = 0.80	M < F
Hojat et al. (2001) [2]	USA	193	4 factors: Physician’s view from the patient’s perspective, Understanding patients experiences feelings and clues, Ignoring emotions in patient care, Thinking like the patient Variance explained: 56 % Factor loadings > 0.46	-	α = 0.89	M < F
Hojat & LaNoue (2014) [24]	USA	2612	3 factors Variance explained: 38 % Factor loadings > 0.25 (except for Item 18)	*χ* ^2^/df = 5.28; AGFI = 0.93; TLI = 0.89; RMSEA = 0.05 Saturation levels > 0.30 (except for item 18) *r* between factors: 0.08 ≤ *r* ≤ 0.78	α =0.80	-
Jumroonrojana & Zartrungpak (2012) [26]	Thailand	708	3 factors	-	α = 0.76	M < F
Leombruni et al. (2014) [27]	Italy	257	-	Modified model: CFI = 0.91; RMSEA = 0.08; WLRM = 0.99 Saturation levels > 0.30 (except for item 18) *r* between factors: 0.24 ≤ *r* ≤ 0.73	α = 0.76	M < F
Magalhães et al. (2011) [28]	Portugal	476	3 factors Variance explained: 37.4 % Factor loadings >0.30 (except for Item 18 and 19)	Modified model: *χ* ^2^/df =1.3; TLI = 0.94 CFI = 0.95; RMSEA = 0.03 (0.05) Saturation levels: not reported *r* between factors: not reported	α = 0.77	
Paro et al. (2012) [29]	Brazil	299	3 factors: CC (11 items), SPS (2 items), PT (7 items) Variance explained. 45 % Factor loadings > 0.35 (except for items 1 and 18: 0.30 and 0.34, respectively)	-	α = 0.84	M < F
Preusche & Wagner-Menghin (2013) [30]	Germany	557	4 factors: PT (11 items), CC (4 items), SPS (2 items), other (4 items) Variance explained: 48 % (forcing 3 factors, variance explained: 36 %) Factor loadings > 0.40	-	α = 0.82 Test-retest: 0.45	-
Rahimi-madiseh et al. 2010 [31]	Iran	181	3 factors: CC (7 items), PT (6 items), STS (3 items) Variance explained: 38 % Factor loadings > 0.49 (item 4, 5, 18 and 19 did not show statistically significant loading)	-	α CC:0.71 PT:0.73 SPS:0.51	M < F
Tavakol et al. (2011) [32]	UK	853	3 factor: CC (10 items), PT (4 items), Emotional detachment (3 items) Variance explained: 42 % Factors loaded in different factors when compared to the original JSE: (items 1, 8 and 15 had no significant factor loadings and were excluded)	Modified model (17 items): *χ* ^2^/df = 1.77; GFI = 0.97; CFI = 0.95; RMSEA = 0.03 Saturation levels > 0.30 (except for item 5) *r* between factors: 0.43 ≤ *r* ≤ 0.75	α = 0.76	M < F
Wen et al. (2013) [33]	China	753	3 factors Variance explained: 48 % Factor loadings > 0.47 (except for item 18, which had n.s. loading)	-	α = 0.83	M < F

Consistent with Hojat’s et al. [2, 5, 23] definition of the construct, researchers using the JSE-S often report and compare the global score of the JSE-S over the three dimensional scores [22, 34–39]. Nonetheless, factorial analysis yielding a more reasonable “correlated multi-factorial model” suggests that empathy is a multidimensional construct [40]. Thus, a total score of the JSE-S relies on the assumption that empathy is a latent second order concept that is manifested through the sub-dimensions of empathy yield by the factorial analysis. Previously reported moderate to strong statistically significant correlations between the three dimensions [27, 32] reinforce this possibility [40, 41]. Since a “correlated multi-factorial model” is not a measurement model per se, as there is not a common target dimension (i.e. empathy) that directly affects items’ variance [40], the test of a second-order model considering and supporting the use of the global JSE-S is needed. Such model, yet to be tested, “places a measurement structure onto the correlations among factors” (p. 4) [40], assuming that the scale’s dimensions share a common cause (i.e. empathy) which explains their correlation.

One translation into Spanish of the JSE-S was developed and tested with Mexican students by Alcorta-Garza and colleagues [25]. The authors translated and back-translated into English the JSE-S questionnaire and assessed its psychometric properties in a sample of 1022 undergraduate medical students. Findings supported this version’s reliability and construct validity. Alcorta-Garza and colleagues’ version has been used to evaluate medical students’ attitude towards empathy in other Spanish speaking countries, including a preliminary study conducted in Spain to assess the impact of a communication skills workshop [41]. Nonetheless, the psychometric properties of the JSE-S in Spanish medical students are unknown. Given Mexico and Spain’s cultural differences, the adaptation and study of the psychometric properties of the JSE-S with Spanish students is essential to assure the validity and reliability of the measure in this population. Such a study is rather relevant to enable the rigorous development of empathy studies in Spain [29, 42], and allow cross-cultural comparisons in medical education research, granting both generalizability of findings and investigation of differences within and between populations. As Portuguese and Italian versions of the JSE-S already exist [27, 28], the availability of a Spanish version would address country and cultural specificities (e.g. South European versus Anglo-Saxon countries) in empathy in patient care interactions, and in empathy evolution during medical training.

The purpose of this study was to: (1) assess the psychometric properties of the Spanish version of the JSE-S in a sample of Spanish undergraduate medical students; (2) test a second order latent factor model for the global JSE-S. Based on previous literature on the validity and reliability of the JSE-S, we predicted that: (1) items would show adequate sensitivity; (2) a factor analysis of JSE-S would yield a three factor solution; (3) a second order confirmatory factor analysis would confirm the existence of a second order latent factor; (4) the scale would present acceptable convergent validity; (5) the internal consistency and composite reliability for the JSE-S and for each sub-scale would be acceptable to good; and (6) empathy of female students would be higher than their male counterparts.

## Methods

### Participants

The population of Spanish medical students in the year 2014/2015 comprised 38765 students. Participants were undergraduate medical students enrolled at the University of Barcelona (a public university) and the International University of Catalonia (a private university) in the year 2014/2015. Inclusion criteria included: (1) being at least 18 years old or; (2) attending the first through sixth year of medical school; and (3) being willing to participate. Of a population of 1502 students attending the 1st through 6th year of medical training in the University of Barcelona and in the International University of Catalonia (65 % women), 1104 students agreed to participate and were included in our sample. Our sample comprised 1024 participants, 689 of which were enrolled at the University of Barcelona and the remaining 415 attended the International University of Catalonia. Table 2 presents the sample’s characteristics. Most students were female (68 %) and age ranged from 18 to 44 (*M* = 20.7, *SD* = 2.59). Most participants were in their first through third year of medical school (61 %), the pre-clinical period of medical training. Average response rate for the total sample was 74 %, with response rate by school and year ranging from 45 % (fifth year) to 100 % (first year) in the International University of Catalonia (IUC), and 56 % (fifth year) to 95 % (first year) in the University of Barcelona (UB). The proportion of female and male participants in our sample (68 % and 32 %, respectively), although approximate to the proportion found in the population of Spanish medical students in the corresponding year (65 and 35 %), was statistically significantly different (*χ*^2^[1] = 5.77, *p* = 0.02).

**Table 2:** Population and sample: proportion of students by gender and year of medical training

		Population of students in the UB and in the IUC		Total sample			Population of students in the UB		UB’s sample			Population of students in the IUC		IUC’s sample		
		*N*	%	*N*	%	Response rate (%)	*N*	%	*n*	%	Response rate (%)	*N*	%	*n*	%	Response Rate (%)
Total		1502	-	1104	-	73.5	938	-	689	-	73.5	564	-	415	-	73.6
Sex	Male	525	35.0	349	31.6	66.5	301	32.1	190	27.5	63.1	224	39.7	159	38.3	71.0
	Female	977	65.0	755	68.4	77.3	637	67.9	499	72.5	78.3	340	60.3	256	61.7	75.3
Year of medical training	1st	273	18.2	263	23.8	96.2	171	18.2	162	23.5	94.7	102	18.1	101	24.3	98.9
	2nd	259	17.2	209	18.9	80.6	158	16.8	120	17.4	75.9	101	17.9	89	21.4	87.9
	3rd	260	17.3	203	18.4	78.1	158	16.8	109	15.8	68.9	102	18.1	94	22.7	92.4
	4th	254	16.9	183	16.6	72.1	156	16.6	95	13.8	61.0	98	17.4	88	21.2	89.8
	5th	238	15.8	123	11.1	51.7	143	15.2	80	11.6	55.9	95	16.8	43	10.4	45.4
	6th	218	14.5	123	11.1	56.6	152	16.2	123	17.9	81.1	66	11.7	-	-	-

### Measures

Participants were asked to provide basic demographic and academic information (sex, age, year of medical training, and university entrance score). Students also completed the adapted Spanish version of the JSE-S. The JSE-S is a 20-item self-report questionnaire assessing students’ attitude towards empathy in the patient-care context. The original JSE-S comprises three domains: *Perspective Taking* (PT), *Compassionate Care* (CC), and *Standing in the Patient’s Shoes* (STS). Participants are asked to report their degree of agreement with each item in a seven-point Likert-type scale, where 1 = “Strongly disagree” and 7 = “Strongly agree”. Three partial scores (PT, CC and STS) and one total score may be computed (by the sum of its corresponding items), with higher scores (ranging from 20 to 140 for the total scale) reflecting higher attitude towards empathy.

Previous findings support the validity and reliability of the original and translated JSE-S [2, 5, 13, 23, 27, 30]. Alcorta-Garza and colleagues tested their Spanish version of the JSE-S in a sample of Mexican medical students, showed adequate internal consistency (alpha = 0.74), and the exploratory factor analysis yielded a three factor structure [25].

### Procedures

The items of the Alcorta-Garza and colleagues’ Spanish version of JSE-S [25] were reviewed by a panel of four European Spanish native speakers, experts in medical education. Minor idiomatic adjustments were carried out in different items in order to correct the idiomatic differences between the idiom in Spain and Mexico. The adjustments were consensual. The Alcorta-Garza and colleagues’ Spanish version of JSE-S and the adapted version used in this study are shown in Additional file 1.

A non-probabilistic sample of participants was recruited between September 2014 and May 2015. Students meeting the inclusion criteria were invited to participate by one of the researchers in person at the end of scheduled class time at the beginning of the academic year (first year students), at the beginning of the second semester (second through fifth year students), or at the end of medical training (sixth year students). Students were specifically informed of the study aims, that participation was voluntary and that responses would be kept anonymous and confidential. Students willing to participate provided oral consent and completed paper-and-pencil versions of the study measures. Students unwilling to participate left the room before the completion of the questionnaire and/or at any point of the questionnaire’s completion. These students were excluded from the sample. There was no set time limit to answer the forms.

Research in medical education in our jurisdiction is exempted from formal approval from the university’s Ethical Committee on the ground that this type of research does not have the purpose to answer a research question on health or biomedicine, does not imply any procedure or intervention that deserves the need for a formal ethical approval, and that the study followed the ethical guidelines regarding the collection of informed consent and anonymity of data processing, in accordance with the ethical Declaration of Helsinki. This study was confirmed as exempt from formal ethical approval by the Ethics review board of the University of Barcelona – Clinical Hospital Medical School Ethical Research Committee.

#### Data analysis

Descriptive statistics (means, standard deviations, medians, skewness and kurtosis) were used for the adapted version of the Spanish JSE-S and the individual items. Items sensitivity was assessed through skewness (Sk) and kurtosis (Ku) analysis, with absolute values higher than three and 10, respectively, indicating severe deviance from normal distribution of the items [43, 44].

The hypothesized three-factor model for the JSE-S was tested through a confirmatory factor analysis (CFA). Model quality of fitness was assessed using the Chi Square (*χ*^2^/df), Comparative Fit Index (CFI), Parsimony Comparative Fit Index (PCFI), Goodness of Fit Index (GFI), Parsimony Goodness of Fit Index (PGFI), and Root Mean Square Error of Approximation (RMSEA). The model was considered to have acceptable or good fit, respectively, if *χ*^2^/df was less than 5 or 2 [45], CFI was higher than 0.8 and 0.9 [46], GFI was higher than 0.9 or 0.95 [47], PCFI and PGFI were higher than 0.6 or 0.8 [48], and RMSEA was lower than 0.08 or 0.05 [47].

Convergent validity was assessed by computing the Average Variance Extracted (AVE) [49]. According to Hair and colleagues’ reference values, AVE higher than 0.5 were suggestive of adequate convergent validity [50].

Given the moderate to strong association between factors found in previous research, and since the JSE total score is many times used in medical education research field, we tested a second order latent factor model, considering the global JSE-S [40, 51]. The model’s adjustment was performed step-by-step, through the analysis of correlation among errors, according to Modification Indices (MI) higher than 11 (*p* < 0.001) [49]. The Chi Square difference test and Expected Cross-Validation Index (MECVI) were computed to compare fit of the initial and final models after adjustments, with statistically significant Chi Square statistic and lower MECVI reflecting better fit [47].

Cronbach’s alphas were computed for the total JSE-S and for the three subscales to assess internal consistency of the scale and its domains. Composite reliability (CR) was also determined [50, 52]. Cronbach’s alpha and CR higher than 0.6 and 0.7 were considered acceptable and good, respectively [43, 53].

Finally, in order to detect interaction effects between gender and year of medical training, as well as gender and year of medical school main effects on empathy ratings, we computed a two-way analyses of variance (ANOVA), with JSE-S as the dependent variable, and gender and year of medical school as the independent variables. Prior to these analyses, we evaluated test assumptions, namely normality and homogeneity of variances, by analyzing Sk and Ku, with absolute values of Sk and Ku lower than three and 10 indicating absence of severe violation of normality assumption [44], and Levene’s test, respectively. JSE-S total scores presented normal distributions for both men and women and for each year of medical training (Sk <1 and Ku <1), and results for the Levene’s test showed no violation of the assumption of homogeneity of variances (*F*_(11,1091)_ = 1.79, *p* = 0.052). In the event that a significant class (year) effect was found, we planned to perform between-temperature comparisons using post hoc Bonferroni tests.

Statistical analyses were computed using software IBM SPSS Statistics (v. 22) and AMOS statistical package (v. 21). Alpha was set at 0.05 for all analyses.

## Results

### Descriptive information

Table 3 shows descriptive statistics for JSE-S total score and for JSE-S individual items for the total sample. The seven-point Likert-type scale was entirely used for all items of the questionnaire, with answers ranging from one to seven. All data generated or analyzed during this study are ﻿included in Additional file 2. With one exception (item 2), items present acceptable skewness (ranging between −2.71 and 0.35; mainly negatively skewed) and kurtosis (ranging between −0.77 and 7.85; mainly leptokurtic) values. The average scores for JSE-S items ranged between 3.67 (*SD* = 1.75) for item 18 and 6.65 (*SD* = 0.72) for item 2.

**Table 3:** Descriptive statistics for JSE-S and for JPSE-S individual items

	M	SD	Me	Min	Max	Sk	Ku
JSE-S Total	117.56	10.43	119.00	50.00	140.00	−1.04	2.90
Item1	6.20	1.34	7.00	1.00	7.00	−2.06	4.00
Item2	6.65	0.72	7.00	1.00	7.00	−3.11	14.46
Item3	4.59	1.32	5.00	1.00	7.00	−0.12	−0.59
Item4	6.30	1.11	7.00	1.00	7.00	−2.04	4.61
Item5	4.57	1.65	5.00	1.00	7.00	−0.36	−0.54
Item6	4.50	1.53	5.00	1.00	7.00	−0.29	−0.77
Item7	6.16	1.22	7.00	1.00	7.00	−1.80	3.17
Item8	5.96	1.26	6.00	1.00	7.00	−1.45	1.91
Item9	6.13	1.17	7.00	1.00	7.00	−1.50	2.11
Item10	6.29	0.98	7.00	1.00	7.00	−1.68	3.26
Item11	6.26	1.09	7.00	1.00	7.00	−1.97	4.56
Item12	6.07	1.18	6.00	1.00	7.00	−1.59	2.50
Item13	6.19	1.01	6.00	1.00	7.00	−1.50	2.62
Item14	6.52	0.87	7.00	1.00	7.00	−2.43	7.24
Item15	6.22	1.06	7.00	1.00	7.00	−1.77	3.85
Item16	6.49	0.82	7.00	1.00	7.00	−2.13	6.32
Item17	5.73	1.32	6.00	1.00	7.00	−1.07	0.74
Item18	3.67	1.75	4.00	1.00	7.00	0.35	−0.76
Item19	6.55	1.00	7.00	1.00	7.00	−2.71	7.85
Item20	6.50	0.87	7.00	1.00	7.00	−2.23	6.09

### Construct validity: confirmatory factor analysis and convergent validity

All of the six combined fit indices for the CFA supported the three factor structure found for the original JSE-S. Three out of six fit indices indicated acceptable fit (*χ*^2^/df = 3.34; PCFI = 0.79; PGFI = 0.76), and the remaining three suggesting good model fit (CFI = 0.90; GFI = 0.95; RMSEA[HI95%] = 0.05[0.05]) (cf. Table 4).

**Table 4:** Model fit indexes for confirmatory factor analysis and second order latent variable analysis

	Number of variables	Number of parameters estimated	*χ*2(df), *p*-value	*X* ^2^/df	CFI	GFI	PCFI	PGFI	RMSEA	MECVI	AIC	Δχ2(df), *p*-value
Model for the CFA	43	Weights: 17	557.18 (167), *p* < 0.001	3.34	0.9	0.95	0.79	0.76	0.046 (*p* = 0.938)	0.59	643.18	
		Variances: 23							95 % C.I.			
		Covariances: 3							[0.043,0.051]			
2nd order latent variable model	47	Weights: 19	557.18 (167), *p* < 0.001	3.34	0.9	0.95	0.79	0.76	0.046 (*p* = 0.938)	0.59	643.18	120.18 (1), *p* < 0.001
		Variances: 24							95 % C.I.			
		Covariances: 0							[0.043,0.051]			
Modified 2nd order latent variable model	47	Weights: 19	437.01 (166), *p* < 0.001	2.63	0.93	0.96	0.82	0.76	0.038 (*p* = 1)	0.48	525.01	
		Variances: 24							95 % C.I.			
		Covariances: 1							[0.035, 0.044]			

**Fig. 1:**
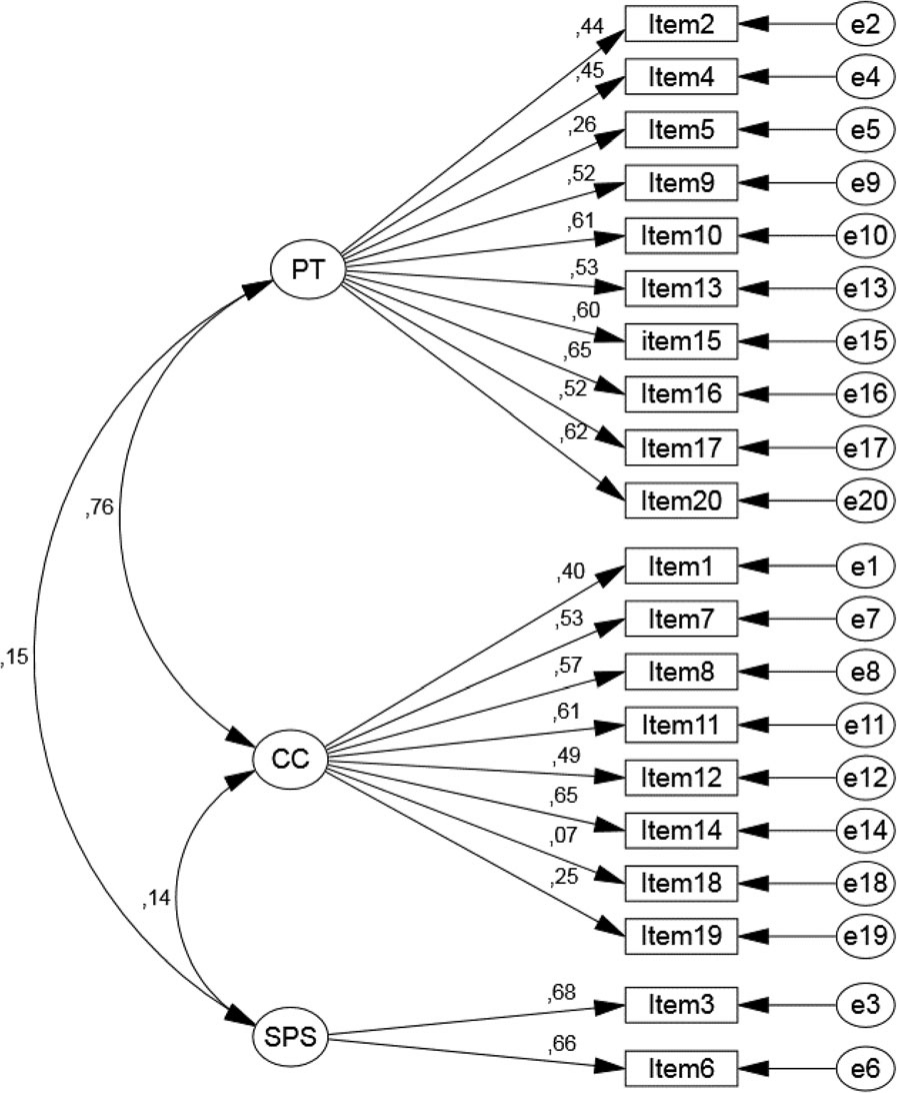
Model for the confirmatory factor analysis

The standardized factorial weights and individual items reliability for the model are presented in Fig. 1. Nine items showed loadings lower than the reference value of 0.50, indicating that less than 25 % of the result of those items were explained by the latent dimension. Yet, 18 out of 20 items exhibited loadings higher than 0.25. Item 18 showed a particularly low saturation level (λ_ij_^2^ = 0.07).

Convergent validity was assessed through AVE. For all three subscales and for total JSE-S the AVE was lower than 0.50. AVE ranged from 0.23 for *Compassionate Care* subscale to 0.45 for *Standing on the Patients Shoes* subscale (see Table 5).

**Table 5:** Average variance extracted and reliability analysis

	JSE-S	PT	CC	SPS
AVE	0.28	0.28	0.23	0.45
CR	0.87	0.79	0.67	0.62
Cronbach’s alpha	0.78	0.76	0.62	0.62
Cronbach’s alpha if item deleted				
Item 1	0.77	-	0.59	-
Item 2	0.77	0.75	-	-
Item 3	0.78	-	-	.
Item 4	0.76	0.75	-	-
Item 5	0.78	0.79	-	-
Item 6	0.78	-	-	.
Item 7	0.76	-	0.57	-
Item 8	0.76	-	0.55	-
Item 9	0.76	0.73	-	-
Item 10	0.76	0.73	-	-
Item 11	0.76	-	0.56	-
Item 12	0.76	-	0.58	-
Item 13	0.76	0.74	-	-
Item 14	0.76	-	0.57	-
Item 15	0.76	0.73	-	-
Item 16	0.76	0.73	-	-
Item 17	0.76	0.73	-	-
Item 18	0.80	-	0.69	-
Item 19	0.78	-	0.62	-
Item 20	0.76	0.73		-

### Second order latent factor model

The second order latent factor model considering the global JSE-S was tested. Since the number of parameters to estimate was the same as the above mentioned modified model, resulting in equal number of degrees of freedom (167), this model presented exactly the same combined fit indexes as the CFA model, suggesting acceptable to good fit.

**Fig. 2:**
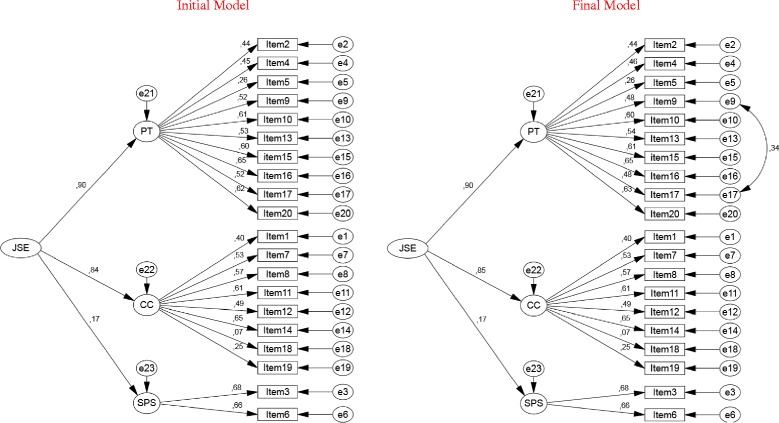
Second order latent factor model

The inspection of JSE-S items suggests that some items have similar content, as for example item 9 (“I try to imagine myself in my patients’ shoes when providing care to them”) and item 17 (“I try to think like my patients in order to render better care”). Based on the analysis of the modification indexes, specific error terms of these items were correlated, resulting in a new modified model that maintained all the items of the original scale (see Fig. 2). Three out of six combined fit indexes suggest good fit of the final model (CFI = 0.93; GFI = 0.96; RMSEA[HI95%] = 0.04[0.04]), while the remaining were suggestive of only acceptable fit (*χ*^2^/df = 2.63; PCFI = 0.82; PGFI = 0.76). The final model presented a goodness of fit higher than the initial one, better than the one found for the initial model (Δχ^2^[1] = 120.18, *p* < 0.001; MECVI: 0.59 vs. 0.48).

The standardized factorial weights and individual items reliability for the initial and final models are presented in Fig. 2. The SPS first order latent variable presented a loading of 0.17, lower than 0.25, and items’ loadings were similar to those found in the CFA model presented above.

### Reliability: internal consistency and composite reliability

The internal consistencies (Cronbach’s alphas) and composite reliability (CR) for the total JSE-S and its dimensions are listed in Table 4. The overall scale, as the subscales, evidenced acceptable to good reliability (JSE-s: α = 0.78, CR = 0.87; TP: α = 0.76, CR = 0.79; CC: α = 0.62, CR = 0.67; SPS: α = 0.62, CR = 0.62) [43, 53]. Values if single items are deleted are comparable to the overall and subscales’ alphas, suggesting that items did not detract from the reliability of the measure, with, maybe, the exception of item 18.

### Gender and year of medical training comparison

Students’ global scores in the JSE-S ranged between 50 and 140 (*M* = 117.56, *SD* = 10.43), with average scores ranging from 113.34 (*SD* = 12.55) and 120.06 (*SD* = 8.46) for male students attending the third and sixth years respectively, and 115.8 (*SD* = 11.48) and 119.88 (*SD* = 9.41) for female students attending the fifth and the second years respectively (see Table 6). No significant interaction effects were found between gender and class (F_(5, 1091)_ = 1.78, *p* =0.119, *η*^*2*^_*p*_ = 0.008, π = 0.61). Significant sex main effects were obtained (F_(5, 1091)_ = 3.73, *p* =0.002, *η*^*2*^_*p*_ = 0,017, π = 0.94), with females (M = 118,6, SD = 9,64) tending to score higher than males (M = 115.4, SD = 1.69). There were also significant year of medical training main effects (F_(1, 1091)_ = 15.88, *p* < 0.001, *η*^*2*^_*p*_ = 0.014, π =0.98), sixth year students show higher empathy levels than first and fifth year students (*p*’s <0.05)

**Table 6:** Average JSE-S total score by gender and year of medical training

Sex		Year of medical training						
		1	2	3	4	5	6	Total
Male	M	114.2	116.7	113.3	116.6	113.5	120.1	115.4
	SD	12.07	12.68	12.55	9.47	11.72	8.46	11.69
Female	M	117.7	119.9	119.7	118.5	115.8	119.5	118.6
	SD	9.19	9.41	9.50	9.39	11.48	9.13	9.64
Total	M	116.7	118.9	117.2	117.9	115.1	119.7	117.6
	SD	10.19	10.61	11.21	9.43	11.56	8.92	10.43

.

## Discussion

The results suggest that the sensitivity, construct validity and reliability of the Spanish JSE-S were acceptable. The convergent validity and individual item sensitivity (item 2) and reliability (item 18) were limited. Even so, findings support the use of the Spanish JSE-S with Spanish medical students. Considering that previous studies supported the validity and reliability of the JSE-S, this measure may be used in cross-cultural studies on medical students’ empathy.

The psychometric sensitivity of the scale and of most items was acceptable. Consistent with previous research in Italy [27], skewness and kurtosis absolute values for the JSE and for individual items were in the range proposed by Kline [44], except for item 2 (“*Patients feel better when their physicians understand their feelings*”). In fact more than 50 % of the participants strongly agreed with the item. The ceiling effect is understandable considering the item’s content, as it is reasonable to expect that most people would be more comfortable whenever their feelings are comprehended by others. Item’s 2 lack of sensitivity, while explicable, redounds in its lower relevance.

The confirmatory factor analysis corroborated that the three-factor structure proposed by the authors of the original version has an adequate fit. The results for item reliability revealed that the factor regression weights for some factors were acceptable and within the range of previous findings [5, 27, 54]. However, these loadings were lower than those of the original JSE. Item 18 showed particularly low and non-significant saturation level, consistent with previous results found for the Portuguese (Brazil), Italian, Spanish (Mexico) and Chinese versions [25, 27−33]﻿, and also in a recent study assessing the factor structure of the JSE-S in the USA [24]. Differences in data analysis - confirmatory factor analysis (a reflective model) versus principal component analysis (a formative model) - might have contributed to these differences. However, other reports on several versions of the measure have identified problematic items (e.g. item 18) suggesting that cross-cultural research would benefit from a modified JSE.

AVEs were lower than the reference values proposed by Hair and colleagues [50], suggesting that the scale has limited convergent validity. Such finding supports the use of the JSE-S total score instead of the measure’s partial ones.

As hypothesized, the Spanish JSE-S and its dimensions showed acceptable to good internal consistency and composite reliability [43, 53], on the range of those found in other translated versions of the measure (0.74 < α < 0.83) [22, 25–28, 30, 32, 33]. Yet, the Cronbach’s alpha of CC if item 18 is deleted is higher than the internal consistency of this dimension, suggesting that this item detracts from the reliability of the subscale. Hence, the eventual elimination of items 2 and 18 could contribute to the improvement of Spanish JSE-S’s psychometric properties, suggesting the convenience to continue the study of the Spanish JSE-S. Both items would benefit from some degree of revision in the near future. While item 2 is more comprehensive, item 18 might have different interpretations and its reformulation needs to be considered. In order to enable future cross-country research, we would recommend the preservation of the original structure of the JSE. Nonetheless, as the structure of the scale is, from the beginning of the original JSE-S development, somewhat unbalanced (the number of items *per* dimension is heterogeneous and two dimensions present only inverted items), only modest internal consistency and construct reliability are, in fact, reasonable to expect.

The tested second order latent factor model presented acceptable to good fit. *Perspective Taking* and *Compassionate Care*, with high regression weights on the second order latent variable, contributed equally and largely than S*tanding in the Patient’s Shoes* to explain the construct of empathy. These results are consistent with the weak inter-scale correlation coefficients found in the confirmatory factor analysis of the correlated multi-factorial model between *Standing in the Patient’s Shoes* with the other two factors. Consequently, our results provide limited support to the use of the Spanish JSE-S total score that assures that empathy is a latent (second order) concept that is manifested through *Perspective Taking*, *Compassionate Care* and *Standing in the Patient’s Shoes*. Such weak correlations are inconsistent with moderate to strong inter-scale correlations found in the Italian and English versions [27, 32]. Hence, our results support the use of the scores for the three dimensions of the Spanish JSE-S over its total score in empathy research in medical education.

As for most of the previous studies using the JSE-S worldwide [27, 35, 37, 39, 55], female students reported significantly higher empathy than their male counterparts, suggesting this version’s ability to detect differences between individuals. Nonetheless, non-statistically significant results have also emerged [36, 56, 57].

Taken together, our findings and previous results from other translated versions, suggest that the validity and reliability of the JSE-S generalize across languages and cultures. Nonetheless, our findings are consistent with previous finding of limited convergent validity, weak inter-scale correlation coefficients, item 2 lack of sensitivity and item 18 low saturation level [24, 25, 27–29]. Such psychometrical limitations reinforce the need to engage in cross-cultural studies comparing: (1) at least, South European countries versions with the Anglo-Saxon countries, and (2) the definition and relevance attributed to the construct of empathy itself across-cultures.

### Limitations

There are a number of limitations that should be taken into account when interpreting the results. First, the cross-sectional design did not allow examination of test-retest reliability and sensitivity of the measure to change. Longitudinal studies are needed to clarify the Spanish JSE-S stability over time, and to assess its ability to detect changes in empathy as a result of interventions. The second relevant limitation regards the generalizability of findings. All students were recruited in only two medical schools in Catalonia Community, and sample was non-probabilistic. The authors are not able to determine how representative the sample is of the population of Spanish undergraduate medical students, as the composition of the sample does not take into account the possible differences between students of different regions of Spain. Third, we did not administer other empathy self-report, patient-report and other-report measures, which would help to further establish convergent validity of the measure. Further research addressing this gap would help to determine the extent of overlap between the adapted version of the Spanish JSE-S and other second and third-person empathy measures.

## Conclusions

The present study is the first, to our knowledge, to assess the Spanish JSE-S psychometric properties in a sample of Spanish medical students. Our findings provide support for the validity and reliability of the adapted version of the Spanish JSE-S with Spanish medical students, confirm the structural validity of the three-factor model, the scale’s satisfactory reliability and ability to discriminate inter-individual differences. Thus, this version may be useful to understand the evolution of empathy in Spanish medical students, as well as in cross-cultural research examining similarities and differences in empathy growth in students from Spain and other countries. Findings provide limited support for the existence of a second order latent factor in the Spanish JSE-S. Based on our findings, it is recommendable that the use of the scores for the three sub-scales of the Spanish JSE-S should prevail over the JSE-S total score.

## Additional files

Additional file 1: Adapted Spanish JSE-S version versus Spanish JSE-S from Alcorta-Garza et al. [24]. (DOCX 14 kb) Additional file 2: DataSheet. (XLSX 107 kb)

## Abbreviations

AVE: Average variance explained
CC: Compassionate care
CFA: Confirmatory factor analysis
CFI: Comparative fit index
CR: Composite reliability
GFI: Goodness of fit index
HI: Confidence interval higher limit
I.C.: Confidence interval
IUC: International University of Catalonia
JSE-S: The Student Version of the Jefferson Scale of Physician Empathy
MECVI: Expected cross-validation index
MI: Modification indices
PCFI: Parsimony comparative fit index
PGFI: Parsimony goodness of fit index
PT: Perspective taking
RMSEA: Root mean square error of approximation
STS: Standing on the patients shoes
UB: University of Barcelona
*χ*^2^/df: Chi square
