# Supplementary material for: Psychometric properties of the Spanish version of the Jefferson Scale of Empathy: making sense of the total score through a second order confirmatory factor analysis
Source: BMC Med Educ. 2016 Sep 19;16:242. doi: 10.1186/s12909-016-0763-5 (PMC5028960; doi:10.1186/s12909-016-0763-5)
Supplement: Additional file 1: — Adapted Spanish JSE-S version versus Spanish JSE-S from Alcorta-Garza et al. [24]. (DOCX 14 kb) [file 12909_2016_763_MOESM1_ESM.docx]

Adapted Spanish JSE-S version *versus* Spanish JSE-S from Alcorta-Garza et al. [24]

|  | **Adapted Spanish JSE-S** | **Spanish JSE-S from Alcorta-Garza et al.** |
| --- | --- | --- |
| **1.** | La comprensión por parte del médico de los sentimientos del paciente y de sus familiares no influye en el tratamiento médico. | Mi comprensión de los sentimientos de mi paciente y sus familiares es un factor irrelevante en el tratamiento médico |
| **2.** | El paciente se siente mejor cuando su médico entiende sus sentimientos. | Mis pacientes se sienten mejor cuando yo comprendo sus sentimientos |
| **3.** | Es difícil para el médico ver las cosas desde la perspectiva del paciente. | Es difícil para mi ver las cosas desde la perspectiva de los pacientes |
| **4.** | La comprensión del lenguaje no verbal es tan importante como la comunicación oral en la relación entre médico y paciente. | Considero que el lenguaje no verbal de mi paciente es tan importante como la comunicación verbal en la relación médico-paciente |
| **5.** | El sentido del humor del médico contribuye a obtener un mejor resultado clínico. | Tengo un buen sentido del humor que creo que contribuye a un mejor resultado clínico. |
| **6.** | Dado que cada persona es diferente, es difícil ver las cosas des desde la perspectiva del paciente. | La gente es diferente, lo que me hace imposible ver las cosas de la perspectiva de mi paciente |
| **7.** | Atender a las emociones de los pacientes no es importante en el momento de hacer la historia clínica. | Trato de no poner atención a las emociones de mis pacientes durante la entrevista e historia clínica. |
| **8.** | Estar pendiente de las experiencias personales de los pacientes no influye en la eficacia de los tratamientos. | La atención a las experiencias personales de mis pacientes es irrelevante para la efectividad del tratamiento. |
| **9.** | El médico debería de intentar ponerse en el lugar del paciente (de ponerse en la piel del paciente) cuando lo está atendiendo. | Trato de ponerme en el lugar de mis pacientes cuando los estoy atendiendo. |
| **10.** | Los pacientes valoran que el médico entienda sus sentimientos, cosa que tiene un valor terapéutico en si mismo. | Mi comprensión de los sentimientos de mis pacientes les da una sensación de validez que es terapéutica por sí misma. |
| **11.** | Las enfermedades de los pacientes solo se pueden curar con tratamientos médicos o quirúrgicos, por tanto, los vínculos emocionales de los médicos con sus pacientes no tienen influencia significativa en los resultados. | Las enfermedades de mis pacientes sólo pueden ser curadas con tratamiento médico; por lo tanto, los lazos afectivos con mis pacientes no tienen un valor significativo en este contexto. |
| **12.** | Preguntar a los pacientes como les va en su vida privada es un factor sin importancia para que el médico comprenda sus molestias físicas. | Considero que preguntarles a mis pacientes de lo que está sucediendo en sus vidas es un factor sin importancia para entender sus molestias físicas |
| **13.** | El médico ha de intentar entender lo que le pasa por la cabeza del paciente fijándose en sus expresiones no verbales y en su lenguaje corporal. | Trato de entender que esta pasando en la mente de mis pacientes poniendo atención a su comunicación no verbal y lenguaje corporal |
| **14.** | Creo que las emociones no tienen cabida en el tratamiento de la enfermedad física. | Creo que las emociones no tienen lugar en el tratamiento de una enfermedad médica. |
| **15.** | La empatía es una habilidad terapéutica y sin ella el médico ve limitados sus éxitos. | La empatía es una habilidad terapéutica sin la cual mi éxito como médico puede estar limitada. |
| **16.** | La comprensión por parte del médico del estado emocional de sus pacientes así como de sus familiares es un componente importante en la relación médico-paciente. | Un componente importante de la relación con mis pacientes es mi comprensión de su estado emocional y el de sus familias |
| **17.** | Los médicos deberían pensar como sus pacientes (intentar ponerse en el lugar de los pacientes) para ofrecer una mejor asistencia. | Trato de pensar como mis pacientes para poder darles un mejor cuidado. |
| **18.** | Los médicos no deberían dejarse influir por vínculos emocionales fuertes con los pacientes y sus familiares. | No me permito ser afectado por las intensas relaciones sentimentales entre mis pacientes con sus familias. |
| **19.** | No disfruto leyendo literatura que no sea médica o de las artes. | No disfruto leer literatura no médica o arte. |
| **20.** | Creo que la empatía es un factor terapéutico importante en el tratamiento médico. | Creo que la empatía es un factor terapéutico en el tratamiento médico. |
